# Tracing Time Trends of Births in Greece

**DOI:** 10.7759/cureus.34040

**Published:** 2023-01-21

**Authors:** Nikolaos Vlachadis, Maria Siori, Georgios Petrakos, Periklis Panagopoulos, Eleni Kornarou, Anastasia Barbouni, Nikolaos Antonakopoulos, Maria Tigka, Aikaterini Lykeridou, Nikolaos Vrachnis

**Affiliations:** 1 Department of Obstetrics and Gynecology, General Hospital of Messinia, Kalamata, GRC; 2 Department of Midwifery, University of West Attica, Athens, GRC; 3 Family Medicine, National Health Service (NHS), Athens, GRC; 4 Department of Obstetrics and Gynecology, National and Kapodistrian University of Athens, Attiko University Hospital, Athens, GRC; 5 Department of Public Health Policy, University of West Attica, Athens, GRC; 6 Department of Public and Community Health, University of West Attica, Athens, GRC; 7 Department of Obstetrics and Gynecology, University of Patras, Patras, GRC

**Keywords:** fertility, time trends, greece, natality, births

## Abstract

Introduction

The aim of this study was to comprehensively investigate and present time trends in births in Greece over the last seven decades.

Methods

Data on live births were derived from the Hellenic Statistical Authority, covering a 72-year period from 1950 to 2021. Trends in the number of births were assessed using joinpoint regression analysis. The annual percentage change (APC) and the average annual percent change (AAPC) were calculated with a 95% confidence interval (95% CI) and level of statistical significance p<0.05.

Results

The overall trend during 1950-2021 was clearly downward (AAPC = -0.9, 95% CI: -1.2 to -0.7). Over the first three decades, births fluctuated to a record high of 162,839 in 1967, with an overall slight downward trend (1950-1981: APC = -0.2, 95% CI: -0.4 to -0.1, p<0.001). During the 1980 decade, the trend was sharply downward (1981-1988: APC = -4.7, 95% CI: -6.2 to -3.2, p<0.001), followed by a stabilization in the 1990s (1988-2001: APC = -0.1, 95% CI: -0.7 to 0.4, p=0.586). The first decade of the 21st century was the only period during the last seven decades with an increasing trend in births in the Greek population (2001-2008: APC = 1.9, 95% CI: 0.3 to 3.5, p = 0.021), but it was followed by plummeting trends during the recent years (2008-2021: APC = -2.7, 95% CI: -3.2 to -2.3, p<0.001), leading to the historic low of 83,756 births in 2019.

Conclusion

The time trend analysis of births in Greece indicated a dramatic plummet in natality in Greece, predominantly attributed to the large decline in births in the 1980s, which could not be reversed in the 1990s and 2000s. The recent decrease in births was associated with the financial recession and has put the Greek population in a disastrous low-fertility spiral.

## Introduction

Over the past seventy years, fertility rates have declined worldwide, and this decrease has been linked to a variety of factors, including the implementation of family planning policies, the improving socio-economic level, and women's empowerment in education and the workforce. Among many countries with declining birth rates, Europe is the continent with the lowest fertility. The European population remained in a long steady state of high natality and mortality, but during the nineteenth century, the demographic transition began with declining mortality rates, especially in the youngest ages, followed by the rapid growth of the population and a reduction in birth rates during the twentieth century. All European countries have moved into the final stage of demographic transition, characterized by low fertility and mortality, and delayed childbearing. In the absence of biological factors explaining the reduced fertility, it is necessary to understand the components of the decline in births in the modern western world [1-4].

Fertility rates are an important area of interest for family planning since, in the context of achieving the reproductive goals of individuals, alongside the promotion of contraceptive methods, monitoring both natality and the treatment of involuntary infertility are required [5]. The recording and study of birth rates is also a central area of research for reproductive medicine. Assisted reproduction technology (ART) has the potential to address demographic challenges and contribute substantially to the rise in births. Advances in reproductive medicine have improved outcomes, and already in many countries, births from the use of ART constitute a substantial proportion of total natality; however, its contribution is limited by the postponement of births to lower fertility ages and the high cost leading to low availability [2].

Greece has one of the lowest birth rates in Europe and is in population decline [4]. The investigation of fertility in the country came to the forefront of the literature in the context of highlighting the negative impact on the Greek population of the unprecedented economic recession that occurred after 2008, and some data on recent downward trends were presented [6-8]. However, detailed data on natality trends in Greece in the previous decades are lacking. The aim of this study was to comprehensively investigate and present time trends in births in Greece over the last seven decades.

## Materials and methods

Official national data regarding live births in Greece based on the birth certificates registered in the country were retrieved from the Hellenic Statistical Authority, covering a 72-year period from 1950 to 2021, the last year with available data.

Year-to-year absolute changes were calculated as the differences between each year's number of births and the number of births of the previous year, and year-to-year relative (%) changes were calculated by dividing each year's absolute change by the number of births of the previous year multiplied by 100.

Trends in the number of births were assessed using joinpoint regression software version 4.7.0.0 (Surveillance Research Program, National Cancer Institute, United States of America). The annual percentage changes (APCs) and the average annual percent changes (AAPCs) were calculated with a 95% confidence interval (95% CI) and level of statistical significance p<0.05. An APC was computed for each time segment between two joinpoints, and an AAPC was used as a summary measure of the trend over a whole pre-specified period, computed as a weighted average of the APCs from the joinpoint model, with the weights in proportion to the length of the APC segment.

Ethics Board approval or consent procedures were not needed since this was an analysis of national-aggregate, publicly available data with no possibility of identifying individuals.

## Results

**Figure 1:**
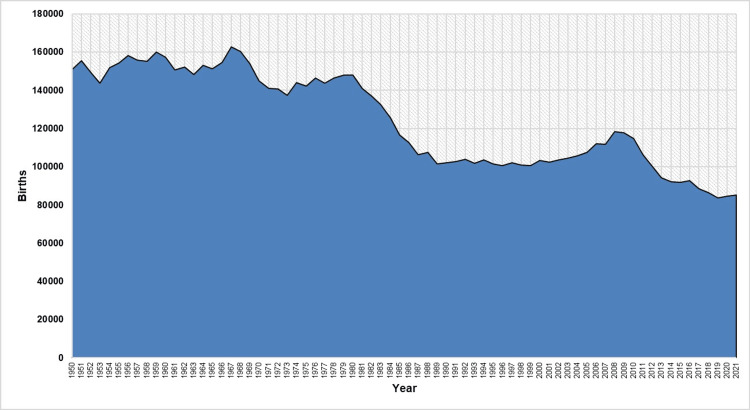
Live births in Greece, 1950-2021

The annual number of live births in Greece during the period 1950-2021 is shown in Figure 1.

In 1950, 151,134 births were registered in Greece, and after fluctuations, the number reached a maximum of 162,839 births in 1967 (an increase of 8%, compared with 1950), after an intermediate rise to 160,199 births in 1960. A marked decline followed, and births reached 137,526 in 1973 (a 16% decrease in six years), only to recover partially again, reaching 148,134 in 1980. Notably, the year 1973 was the only one in the first three decades that births fell below 140,000.

The 1980s was a period of rapid decline in the number of births in Greece, reaching 101,657 in 1989, a 31% drop during 1980-1989. In contrast, the 1990s were characterized by a stabilization of births at a low of just over 100,000, and the last year of the 20th century (1999) recorded a historic 20th-century low of 100,643 births.

In the first decade of the 21st century, births rose to 118,302 in 2008 (an 18% increase since 1999). However, from 2008 onwards, the number of births in the country decreased continuously, with births falling for the first time below 100,000 in 2013 (94,134) and below 90,000 in 2017 (88,553), and finally reaching a historic low of 83,756 in 2019 (a 29% decline or 34,546 fewer births than in 2008), before rising slightly again in the last two years (2020: 84,764, 2021: 85,346). During the last five years (2017-2021), births in Greece ranged below 90,000 births per year.

During the period under investigation, the record-high number of births was in 1967, and the all-time low was in 2019, a 49% relative decrease with 79,083 fewer births. The largest portion of these births was "lost" in the 1980-1989 decade (59% or 46,477 fewer births).

**Figure 2:**
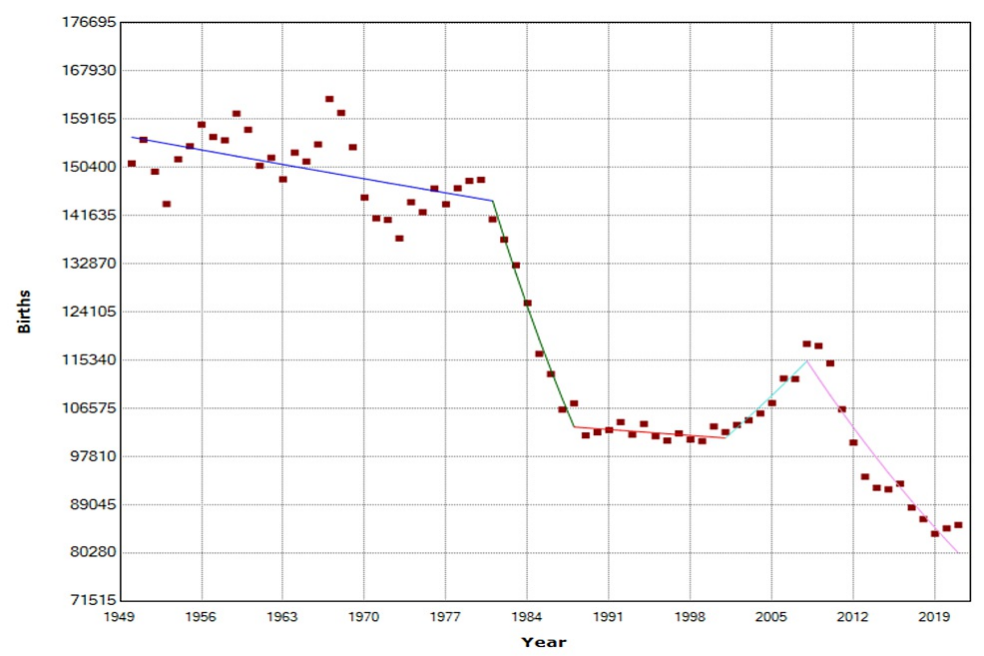
Time trends in births in Greece, 1950-2021

The trends for the 1950-2021 period are presented in Table 1 and Figure 2. 

**Table 1:** Time trends in births in Greece, 1950-2021

Period	APC	95% CI	p-value
1950-1981	-0.2	-0.4 to -0.1	<0.001
1981-1988	-4.7	-6.2 to -3.2	<0.001
1988-2001	-0.1	-0.7 to 0.4	0.586
2001-2008	1.9	0.3 to 3.5	0.021
2008-2021	-2.7	-3.2 to -2.3	<0.001

The overall trend during 1950-2021 was clearly downward (AAPC = -0.9, 95% CI: -1.2 to -0.7). Over the first three decades, an overall slight downward trend emerged (1950-1981: APC = -0.2, 95% CI: -0.4 to -0.1, p<0.001). During the 1980 decade, the trend was clearly downward (1981-1988: APC = -4.7, 95% CI: -6.2 to -3.2, p<0.001), followed by a stabilization in the 1990s (1988-2001: APC = -0.1, 95% CI: -0.7 to 0.4, p=0.586). The first decade of the 21st century was the only period of an increasing trend in births in the Greek population during the last seven decades (2001-2008: APC = 1.9, 95% CI: 0.3 to 3.5, p = 0.021), but it was followed by decreasing trends during the latest years (2008-2021: APC = -2.7, 95% CI: -3.2 to -2.3, p<0.001). 

**Figure 3:**
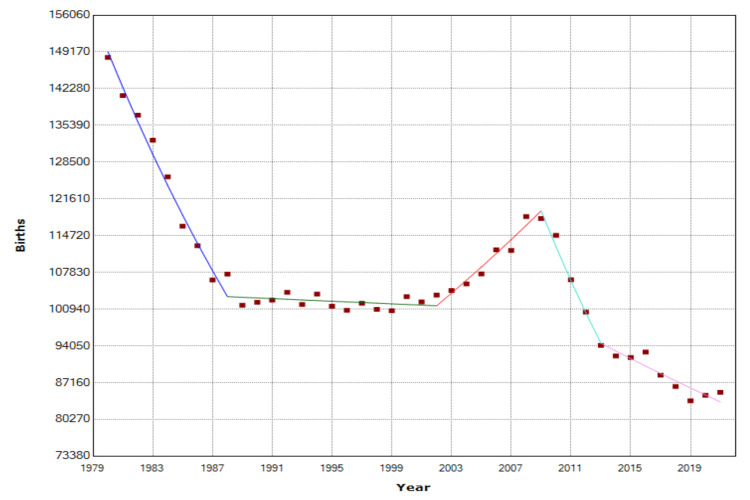
Time trends in births in Greece, 1980-2021

The trends in births in Greece during the most recent period of 1980-2021 are presented in more detail in Table 2 and Figure 3.

**Table 2:** Time trends in births in Greece, 1980-2021

Period	APC	95% CI	P-value
1980-1988	-4.5	-4.9 to -4.1	<0.001
1988-2002	-0.1	-0.3 to 0.1	0.270
2002-2009	2.3	1.6 to 3.1	<0.001
2009-2013	-5.7	-7.6 to -3.7	<0.001
2013-2021	-1.5	-2.0 to -1.1	<0.001

The descending trend in births in the country over these four decades was with AAPC = -1.4 (95% CI: -1.7 to -1.1). The period after 2008 was further divided into two segments: during 2009-2013, the trend showed sharp falling trends (APC = -5.7, 95% CI: -7.6 to -3.7, p<0.001), and the decrease continued, albeit at a lower rate, in the latest period 2013-2021 (APC = -1.5, 95% CI: -2.0 to -1.1, p<0.001).

**Figure 4:**
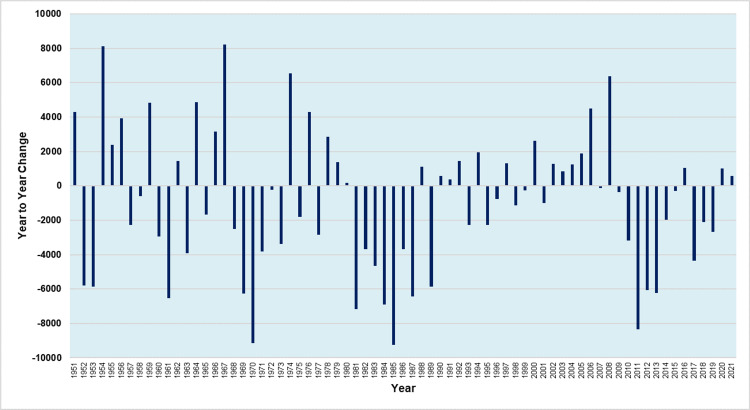
Absolute year-to-year change in the number of births in Greece, 1950-2021

**Figure 5:**
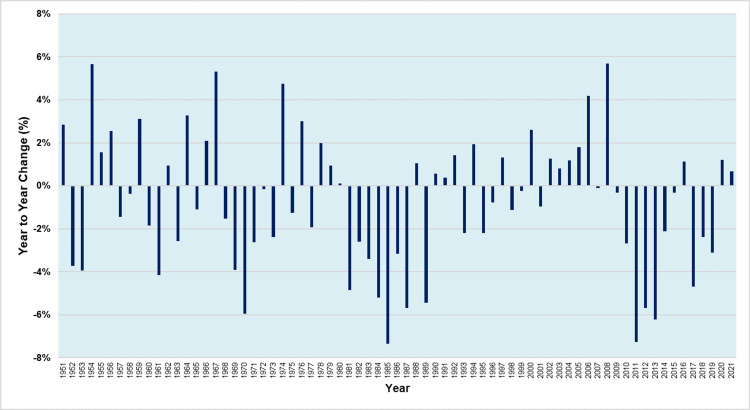
Relative (%) year-to-year change in the number of births in Greece, 1950-2021

Figures 4 and 5 present the year-to-year absolute and relative changes in births in Greece during the period 1950-2021, respectively.

During 1950-2021, there was an annual increase in 30 years and an annual decrease in 41 years.

The period 2002-2006 was the only one with five consecutive years of rising births. There were only five more periods of continuous increase in births, namely three triennia (1954-1956, 1978-1980, 1990-1992) and two biennia (1966-1967 and 2020-2021), whereas the remaining twelve years of increase were isolated (1951, 1959, 1962, 1964, 1974, 1976, 1988, 1994, 1997, 2000, 2008, and 2016), i.e., preceded and followed by years of decline.

The largest absolute annual increase in the number of births occurred in 1967, with 8,226 more births, compared with 1966. This was followed by 1954 (+8,127 births) and 1974 (+6,543 births), while the fourth largest annual increase was in 2008. Moreover, the largest relative annual increase in the number of births was in 2008 and 1954 (+5.7%), followed by 1967 (+5.3%).

Conversely, there were three periods of consecutive year-to-year declines; 1981-1987 and 2009-2015, when Greece saw seven consecutive years of births decline, and 1968-1973 with six years of decrease. The overall reduction in births was 28%, 22%, and 16% for these periods, respectively. Furthermore, there were six more periods of continuous decrease in births, namely one 3-year period (2017-2019) and five biennia (1952-1953, 1957-1958, 1960-1961, 1995-1996, 1998-1999), whereas the remaining eight years of decrease were isolated (1963, 1965, 1975, 1977, 1989, 1993, 2001, and 2007).

The largest absolute annual reduction in the number of births occurred in 1985, with 9,243 fewer births, compared with 1984. This was followed by 1970 (-9,149 births), while the third largest annual decrease was in 2011 (-8,338 births). Moreover, 1985 was also the year of the largest relative annual birth decline (-7.4%), followed by 2011 (-7.3%) and 2013 (-6.2%).

## Discussion

This study presents the trends in the number of births in the Greek population over the last seven decades. The results reveal an overall remarkable decreasing trend in births in Greece. The 1950-2021 birth trends can be divided into five main periods: three periods of decrease, one period of stabilization, and one segment of increase. The three segments of declining natality include the modest decrease in the first three decades, the steep decline in the 1980s, and the latest falling trend that began with the great financial crisis. In contrast, at the beginning of the 21st century, Greece saw the only post-war period with rising birth trends, while birth rates in the country were unchanged during the 1990s.

This study included data on live births in Greece since 1950. In 1947, the Dodecanese islands were incorporated into Greece, and the country acquired its present size, while 1949 saw the end of the civil war in the country [7]. Consequently, 1950 was the first year in which the first official compilation of the number of births after World War II was possible.

In Greece, the 1950s and 1960s were periods of economic growth but also of high migration of young people to Western Europe. The number of births in the country was generally on the rise and reached an all-time post-World War II high of 162,839 births in 1967. Negative socio-political developments, including the imposition of a dictatorial government in Greece, and the intensification of the migratory flow, led to a large decline by 1973, followed by only a slight recovery by the end of the decade [7]. Overall, during the first three decades, 1950-1980, the annual number of births fluctuated steadily above 140,000 (with the exception of the year 1973), with an overall statistically significant descending trend.

A sharp decline in births in the Greek population appeared during the 1980s. Indeed, our analysis showed that during this decade, births in the country fell dramatically by 31% from 1980 to 1989, and the downward trend was with APC = -4.5% in the period 1980-1988. In 1985, the greatest annual absolute and relative decline in births was registered in the Greek population.

The number of births remained unchanged during the 1990s, albeit at very low levels. Probably, this was associated with the return of Greek emigrants from Western Europe, as well as the fact that Greece became a receiving country for immigrants from the former Communist countries of Eastern Europe. The entry of people of childbearing age into the country halted but did not reverse the rapid declining trends in births of the 1980s [7].

The improvement in economic rates, the increase in the number of births to foreign mothers, as well as the emergence of late births by older women who had postponed childbearing in previous years [7] resulted in the first decade of the 21st century in Greece seeing the last upward trend in births (18% during 1999-2008). This period saw the only five-year consecutive increase in births (2002-2006), and 2008 was marked by the largest relative annual increase (+5.7%) after World War II.

The rising trend was abruptly interrupted by the major economic crisis that hit Greece, and the period after 2008 was marked by a dramatic drop in birth rates. Births fell for seven consecutive years (2009-2016), at a rate comparable to that in the 1980s; they recovered temporarily in 2016 and then fell again for three more consecutive years (2017-2019), reaching a record low of 83,756 births in 2019, almost half of those in 1967, and down 29% compared with 2008. The analysis of the 1980-2021 period revealed that the greatest part of the decline in birth rates in the country occurred after 1980 (the AAPC during 1980-2021 was -1.4, whereas APC was -0.2 for the period 1950-1980) and that the economic recession decrease was divided into two periods: a fast decline during the first four years (2009-2013) and a slower decline after 2013. Finally, in the years 2020 and 2021, there was a weak increase in births, despite the Sars-CoV-2 pandemic waves that seem to have negatively affected natality in many countries [9].

This study presents a comprehensive analysis of the time trends in births in Greece, covering the recent seven decades for the first time in the medical literature. However, our analysis is limited to the investigation of the time trends of births in the Greek population, and although the absolute number of births is undoubtedly the main parameter for assessing fertility, further analysis is essential to assess the impact of factors such as the population of women of reproductive age, using the general fertility rate (GFR) and the age-specific birth rates, as well as several socio-economic parameters [10-11]. The association of fertility with economic conditions has been a classic research topic, and although it is generally considered that fertility follows economic cycles, the relationship could be more complicated [2,7]. In Greece, the two steepest declines occurred in two periods with very different economic circumstances: during the 1980s, a period of economic growth after the entrance of Greece into the European Union, as well as after the economic recession of 2008.

The reduction of birth cohorts at a sub-replacement fertility level has profound health and socio-economic implications for the Greek population [12]. Also, in recent decades in Greece, there has been a shift towards older maternal ages, with a decrease in teenage births and those of women under 30 years and an increase in births of women over 35 years of age, which are associated with reduced fertility and increased perinatal complications [13-16]. Noteworthy, Greece has very high preterm birth rates [17-19], and childbearing postponement has led to increased use of fertility treatments. However, ART can have a demographic relevance only when women take advantage of it at a younger age, but currently, it cannot have a significant demographic impact on women of advanced reproductive age [14-20].

## Conclusions

The time trend analysis of births in Greece during the last seven decades indicated that the dramatic plummet in natality in the country is predominantly attributed to the large decline in births in the 1980s, which could not be reversed in the 1990s and 2000s. The recent financial recession has put further pressure on the birth rates of the Greek population, which has entered a disastrous low-fertility spiral. The shrinking birth rate in Greece constitutes a major public health challenge, and further in-depth studies, as well as health policies to enhance fertility, are warranted.
